# Cyanidin-3-glucoside: targeting atherosclerosis through gut microbiota and anti-inflammation

**DOI:** 10.3389/fnut.2025.1627868

**Published:** 2025-06-30

**Authors:** Zihan Tang

**Affiliations:** Hunan Provincial Engineering Research Center of Applied Microbial Resources Development for Livestock and Poultry, College of Bioscience and Biotechnology, Hunan Agricultural University, Changsha, China

**Keywords:** anthocyanins, C3G, gut microbes, atherosclerosis, chronic inflammation, vascular endothelial cells

## Abstract

With the shifting global disease spectrum, atherosclerosis (AS) has emerged as a leading contributor to mortality worldwide, with associated cardiovascular diseases (CVDs) representing the predominant cause of death. AS, a chronic inflammatory pathology, is mechanistically linked to oxidative stress and gut microbiota dysbiosis, which drive excessive reactive oxygen species (ROS) production and elevated levels of pro-inflammatory cytokines. Dietary polyphenols, particularly anthocyanins, are well-characterized for their dual role in modulating gut microbial communities and ameliorating chronic inflammatory conditions. Cyanidin-3-glucoside (C3G), a water-soluble flavonoid abundant in pigmented fruits and vegetables, exhibits potent antioxidant, anti-inflammatory, and anti-hypertensive bioactivities. More importantly, C3G engages in bidirectional interactions with the gut microbiota. It alters microbial composition and undergoes bacterial enzymatic metabolism to generate phenolic derivatives, including protocatechuic acid (PCA), which demonstrate enhanced systemic bioavailability and bioactivity. These metabolites improve endothelial function by augmenting nitric oxide (NO) bioavailability through endothelial nitric oxide synthase (eNOS) activation and regulating lipid homeostasis through ATP-binding cassette transporter G1 (ABCG1)-mediated pathways. Therefore, this review describes the dual mechanistic role of C3G as a phenolic bioactive compound and a prebiotic modulator, highlighting its therapeutic potential in chronic disease prevention through microbiota-dependent and -independent pathways. These insights underscore the need for advanced mechanistic studies to identify specific bacterial taxa involved in C3G biotransformation and to optimize targeted delivery systems to maximize their therapeutic efficacy.

## 1 Introduction

Anthocyanins are flavonoids that are widely found in fruits and vegetables, providing them with color (1), and they are especially abundant in red, blue, and purple berries. There are six types of anthocyanins in edible fruits and vegetables: cornflower, delphinium, geranium, paeoniflorin, petunia and mallow (2). According to many studies, anthocyanins extracted from fruits and vegetables not only can be used as dyes and food coloring agents (2), but they also have medicinal values such as anti-diabetic (3), anti-cancer (4), antibacterial (5), anti-inflammatory (6), and ability to reduce the incidence of obesity, and secondly, anthocyanins have a synergistic effect with combinations of vitamins and minerals in preventing or reducing the risk of macular degeneration (7).

Cyanidins are considered the most widely distributed anthocyanins in the plant kingdom. Cyanidin-3-glycoside (C3G), also known as kuromanin, is one of the most widely studied cornflower glycosides, which are found in the human diet, mainly through vegetables, legumes, fruits, and red wine (8). In recent years, research on C3G has become more extensive and in-depth, and more therapeutic mechanisms and therapeutic potentials of C3G have been developed. C3G can achieve anti-inflammatory effects by inhibiting the protein expression of the intracellular pro-inflammatory cytokines TNF-*α* and IL-1β (9), and protect against acute lung injury (10). Moreover, C3G can upregulate the expression of UCP1 and other heat-producing genes, as well as other heat-producing genes, in the inguinal white adipose tissue and brown adipose tissue; thus, it can be used as an anti-inflammatory agent. In addition, C3G can up--regulate the expression of UCP1 and other thermogenic genes in white adipose tissue and brown adipose tissue in the groin, thus preventing and treating obesity-related complications (11). Some studies have also found that C3G can inhibit the abnormal proliferation of cancer cells through the caspase-3 cleavage of the Bcl-2 and Bax pathways and the occurrence of apoptosis through DNA fragmentation (12).

AS arises from the formation of atheromatous lipid-containing necrotic foci and vascular wall hardening. This process is driven by lipid deposition and accumulation of blood components in the arterial intima, accompanied by smooth muscle cell proliferation and increased collagen fiber production. Pathologically, the disease manifests through narrowed vascular lumen, reduced elasticity, and thickened and hardened vessel walls, with its fundamental characteristics being lipid deposition in the arterial intima and the development of multiple heterogeneous atherosclerotic plaques (13). In addition, many experiments have demonstrated that AS is related to inflammation. Particular, protein fragments derived from cytokines, chemokines, and other immune-related proteins play a central role in regulating inflammatory and immune responses in atherosclerotic plaques (14). An increased abundance of low-density lipoproteins (LDL) causes immune cells to accumulate in the intima, thereby exacerbating the inflammatory response. Chronic inflammation leads to the oxidation of high-density lipoproteins (HDL), which in turn alters the abundance of LDL, while elevated levels of lipids are considered an important risk factor for the development of plaque formation and progression. With the continuous improvement of human living standards, dietary patterns have undergone significant changes, leading to a growing population with dyslipidemia and hypertension. These metabolic disturbances have substantially increased the risk of AS. This pathological process, marked by focal fibrotic thickening of the vascular intima, progressively narrows arteries in various organs, ultimately triggering diseases such as coronary heart disease, aneurysms, ischemic nephropathy, and cerebral infarction (15). Notably, sudden spikes in blood pressure may result in fatal outcomes due to massive hemorrhages. The causes of AS lead to heart attacks and strokes. Currently, despite the rapid development of treatments for cardiovascular disease, the long-term consequences of AS remain the leading cause of death in both developed and developing countries. Due to its rapid progression later in the course of the disease, AS was ranked by the WHO as one of the leading causes of disease mortality as early as 2016. This is despite the wide range of medications currently used in the treatment of cardiovascular diseases, such as statins, fibrates, angiotensin receptor blockers, angiotensin converting enzyme inhibitors, calcium channel blockers, and angiotensin and beta receptor blockers (16). However, it is important to recognize that a large portion of the population has strong adverse reactions to these drugs, which can be life-threatening in severe cases. Therefore, the discovery and cultivation of innovative cardiovascular disease therapeutic strategies with fewer side effects andreal-world applications is an important clinical need. There is a large body of research suggesting that people who eat a balanced diet have a reduced risk of AS compared with those who lack anthocyanin-rich fruits and vegetables in their diets, and that anthocyanins play a role in preventing plaque rupture and subsequent thrombosis in patients with AS.

## 2 Bio-absorption and the metabolic process of C3G

### 2.1 Brief description of C3G

**Figure 1:**
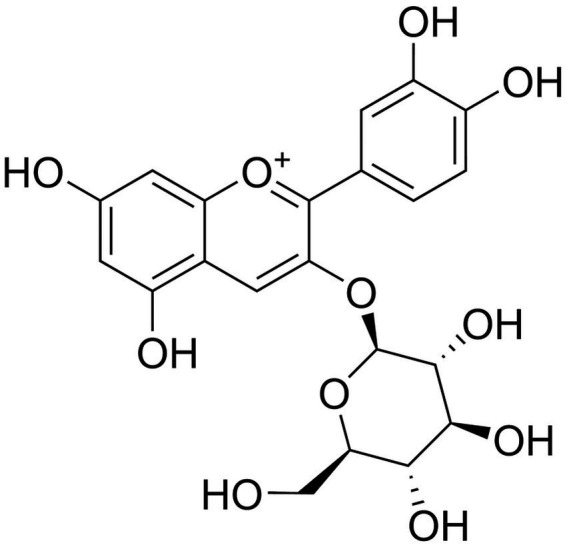
Chemical structure of C3G. C3G has a molecular weight of 449.4 g/mol, and it consists of o-glycosylated anthocyanins with two hydroxyl groups in the third aromatic ring.

C3G is one of the most abundant monomers belonging to anthocyanins, and it belongs to the class of flavonoids. The molecular weight of C3G is 449.4 g/mol. It is glycosylated from the glycosidic element anthocyanidin, which increases the polarity of C3G, thus making C3G more hydrophilic than anthocyanidins (11). The molecular weight of C3G is 449.4 g/mol (Figure 1). It is glycosylated from the glycosidic element anthocyanidin, which increases the polarity of C3G, and thus makes it more hydrophilic than anthocyanidins. In addition, C3G has strong antioxidant activity because it C3G consists of o-glycosylated anthocyanins and because of the presence of two hydroxyl groups in the third aromatic ring (Figure 1). C3G is usually found as a reddish pigment in a variety of vegetables and fruits, but the structure of anthocyanins is easily affected by pH, light, and temperature (1).

### 2.2 Bio-absorption and the metabolism of C3G

**Figure 2:**
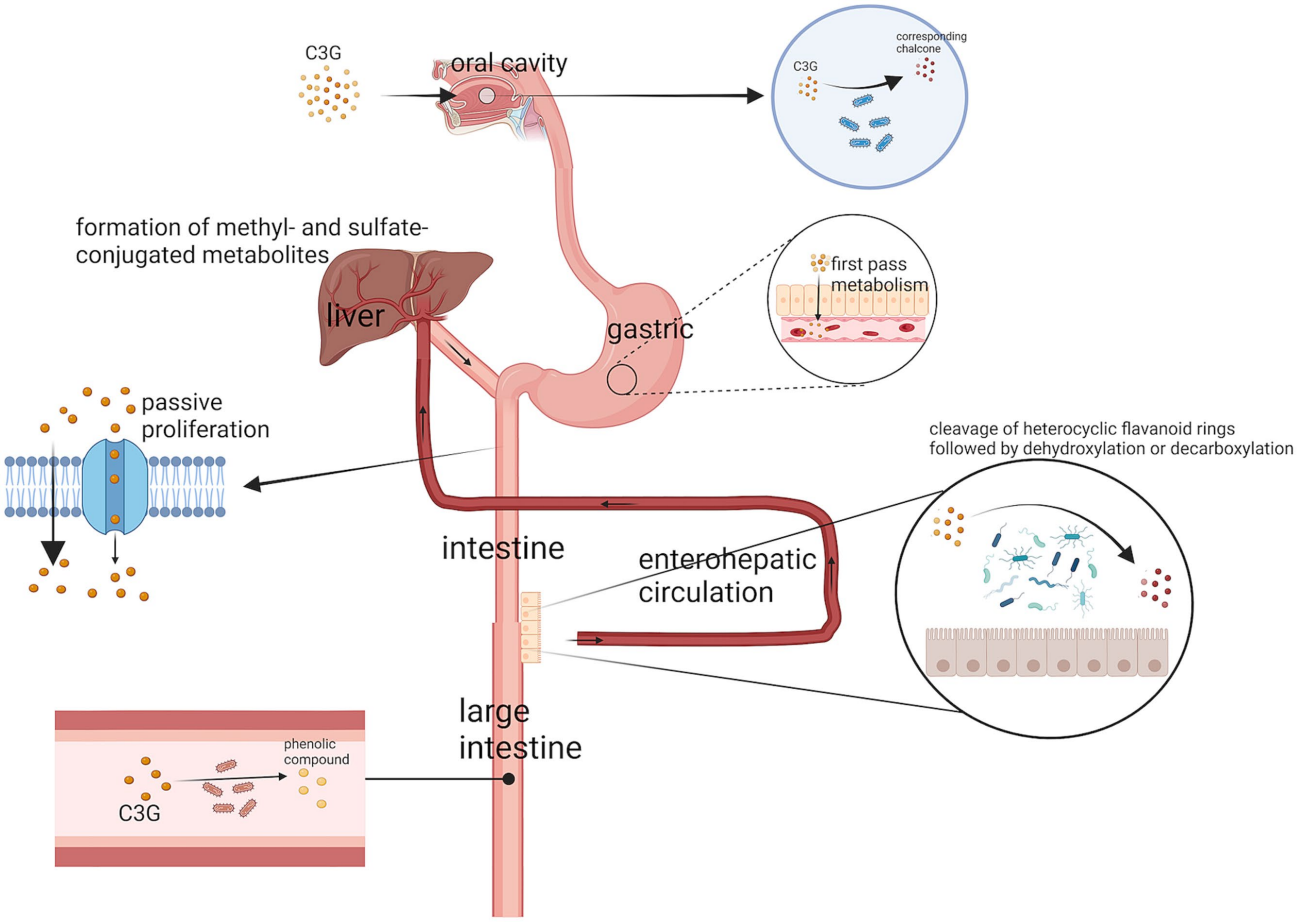
The digestive pathway of C3G. A small portion of C3G is metabolized by microorganisms in the oral cavity to the corresponding chalcone. The stomach then rapidly absorbs a large portion of C3G, while the remaining C3G is absorbed through passive diffusion in the small intestine and broken down by microorganisms in the colon. The metabolites of C3G also enter the liver through the enterohepatic circulation and blood circulation to form methyl- and sulfate-conjugated metabolites.

C3G is first metabolized in the oral cavity (17). Anthocyanins are metabolized by the oral microbiota in the oral cavity and the glycosidic moiety is removed, ultimately converting the anthocyanin into the corresponding chalcone (18). The gastric mucosa is considered one of the major sites of C3G uptake (19). C3G can be efficiently absorbed from the gastrointestinal tract (20), undergoes extensive first-pass metabolism, and enters blood circulation as metabolites. After passing through the gastric region and being rapidly absorbed, C3G enters the small intestine. AsC3G is hydrophilic in nature, most of the C3G can be absorbed directly by the small intestine through passive diffusion, while the remaining C3G is absorbed in the distal small intestine. As C3G is hydrophilic, most of it can be absorbed directly by the small intestine through passive diffusion. The remaining C3G is decomposed by the microbiota in the distal small intestine, such as the ileum, and in the distal large intestine, such as the colon, which plays a major absorption role (21). Subsequently, phase II metabolites and multiphase metabolites of C3G (including bacterial metabolites) can enter the liver and kidneys through the enterohepatic (20) and blood circulations to form additional methyl- and sulfate-conjugated metabolites (22) (Figure 2). In the colon, C3G is broken down by gut microorganisms into a number of simple phenolics or phenolic acids such as gallic acid, protocatechuic acid (22), butyric acid, p-coumaric acid, vanillic acid, cinnamic acid, phenylpropionic acid, or homocoumaric acid, which allow the conversion of C3G into a more bio-available and more readily absorbed form (1). In addition, the absorption of C3G can be affected by food and other flavonoids.

### 2.3 Bioavailability of C3G

Anthocyanins have very low bioavailability and low diffusion rates, and even when ingested in large quantities, their levels in the blood remain low. When rats ingested 500 mg/kg of C3G-rich pterostilbene extract, their blood Cmax of C3G was 69.034 ± 8.051 nM, and their AUC0-10 was 131.314 ± 12.185 nmol h L ^−1^ (23). However, it has been found that through microencapsulation (24) or nanoformulation (25), it is possible to inhibit their breakdown and increase absorption and bioavailability, thus reducing the disadvantages associated with low bioavailability.

## 3 Effects of C3G on inflammation

### 3.1 Inflammation and AS

Traditionally, AS has been considered a cholesterol storage disease (26), in which lipids are deposited within the arterial intima and form many inhomogeneous atherosclerotic plaques. Thus, the lumen of the blood vessel becomes smaller, its elasticity decreases, and the wall thickens and hardens (13). In the process of plaque formation, internal hemorrhage, rupture of plaques, and calcification may also occur, resulting in the formation of thrombus and atheromatous tumors, which ultimately affect the artery’s blood supply and causes peripheral tissue, organ ischemia, or necrosis. However, researchers have found that AS is a chronic inflammatory disease with an autoimmune component. This is evidenced by antibodies against low-density lipoprotein and a number of other atherosclerotic antigens that have been found in all clinically diagnosed patients and in animal models of successful AS modeling (26).

The pathogenesis of AS begins with the accumulation of a few plasma lipoproteins in the sub-endothelial space at sites of disturbed blood flow and endothelial dysfunction, LDL is oxidatively modified by reactive oxygen species (ROS) in the intima of arterial vessels, thereby promoting the uptake of oxLDL by macrophages and the formation of foam cells (1). In addition, oxidized phospholipids trigger arterial wall inflammation by associating with toll-like receptors (TLRs), a group of broadly expressing pattern-recognition receptors (PRRs) that induce pro-inflammatory signals. Natural low-density lipoprotein may also be absorbed into macrophages through microphagocytosis or phagocytosis in the aggregated form of cholesterol compounds or crystals. The continuous inflow of cholesterol ultimately overwhelms the metabolizing ability of phagocytes, forming an intracellular lipids droplet.

Cholesterol loading has been considered to elicit myeloid responses, including the secretion of a number of proinflammatory cytokines, the proliferation of macrophages *in situ*, and the further recruitment of myeloid cells. Cholesterol loading activates inflammatory vesicles that can cleave pro-IL-1β to its biologically activated form. IL-1β acts as an inflammatory master cytokine that enhances the expression of many pro-inflammatory cytokines and CRP22. In addition, macrophage value-addition and activation result in the liberation of hydrolase cytokines, chemokines, and growth factors, which can induce further injury and eventually result in focal necrosis.

### 3.2 Gut microbes and inflammation

#### 3.2.1 C3G affects inflammation by altering the relative abundance of gut microbes

**Figure 3:**
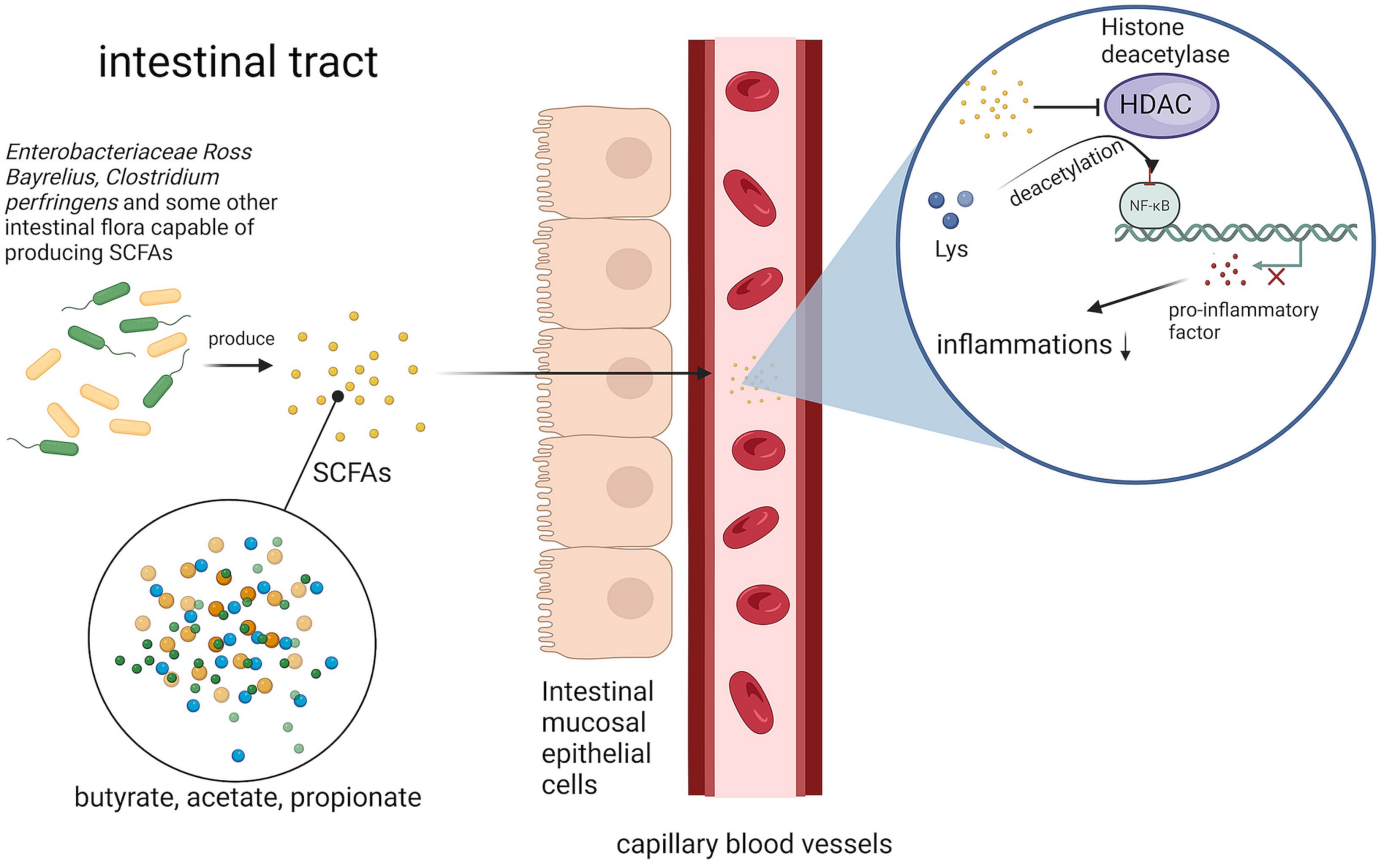
Pathways by which gut microbes attenuate the inflammatory response. Gut microorganisms inhibit butyric acid-mediated HDAC by synthesizing SCFAs, thereby reducing the transcriptional activity of the NF-κB pathway and decreasing the inflammatory response.

In some studies, gut microbiota can be synthesized short-chain fatty acids (SCFAs) metabolites from dietary fibers in the gut; these metabolites (including acetic acid, propionic acid, and butyric acid) can enter the bloodstream (27). Within the circulation, SCFAs inhibit histone deacetylase (HDAC) activity and suppress lysine residue deacetylation. Specifically, butyric acid-mediated HDAC inhibition reduces transcriptional activity of the NF-κB pathway, thereby decreasing macrophage production of pro-inflammatory cytokines and attenuating inflammatory responses (28) (Figure 3).

Fecal samples (representative of the gut microbiome) from 187 healthy controls and 218 AS patients were sequenced and a macrogenome-wide association study (MWAS) was conducted to identify the strains and function modules associated with atherosclerotic cardiovascular disease (29). After comparing the abundance of gut microbes in nominal patients with AS, *Streptococcus* and *Escherichia coli*, which are associated with inflammation, were found to be enriched in patients with AS, while *Enterobacteriaceae Ross Bayrelius* and *Clostridium pallidum*, which synthesize SCFAs, were found to be consumed. The reason for this departure from a healthy state of the gut microbiota of patients with AS is that Enterobacteriaceae and Streptococcus are abundant and may functionally metabolize or transport several molecules essential for cardiovascular health (29).

Anthocyanins can be converted in the gut by gut microbes into more bio-available and more absorbable forms, such as protocatechuic acid (30), which may also modulate the composition of the colonic microbiota. A number of researchers have associated C3G ingestion with an increase in beneficial bacteria such as *Bifidobacteria Lactobacillus* or *Actinobacillus* (22). C3G, which is linked by a *β*-glucosidic bond to glucoside, undergoes enzymatic degradation by β-glucosidase secreted by probiotics. This degradation provides additional energy to support bacterial proliferation. Furthermore, cleavage of the β-glucosidic bond releases the anthocyanidin backbone (2-phenylbenzopyrylium), which is further metabolized into phenolic acids and SCFAs. These metabolites lower the environmental pH, creating an acidic intestinal milieu that favors probiotic growth. Beyond the direct effects of C3G on probiotics such as *Bifidobacterium* and *Lactobacillus*, its microbial metabolite phenyllactic acid also contributes to intestinal homeostasis (31). Phenyllactic acid inhibits the growth of fungi and pathogenic bacteria (e.g., *Staphylococcus aureus* and *E. coli*) (32), thereby reshaping the gut microenvironment to enhance probiotic colonization. This bidirectional interaction between C3G and gut microbiota establishes a synergistic loop for improving intestinal health. Probiotic bacteria can have many positive effects on the body’s health; thus, the observed positive impact after anthocyanin intake may be partly attributed to the regulation of the intestinal microbiota.

One of the mechanisms by which anthocyanins increase the number of probiotics in the gut is the production of SCFAs. The gut bacterial metabolism of anthocyanins disrupts the glycosidic bond, which produces SCFAs (33) and phenolic acids (31), both of which lead to a decrease in pH and create an environment that stimulates the proliferation of probiotic bacteria. The metabolism of anthocyanins is also known to increase the number of probiotics in the gut, which is a key factor in the increase in the number of probiotics in the intestine (34).

**Figure 4:**
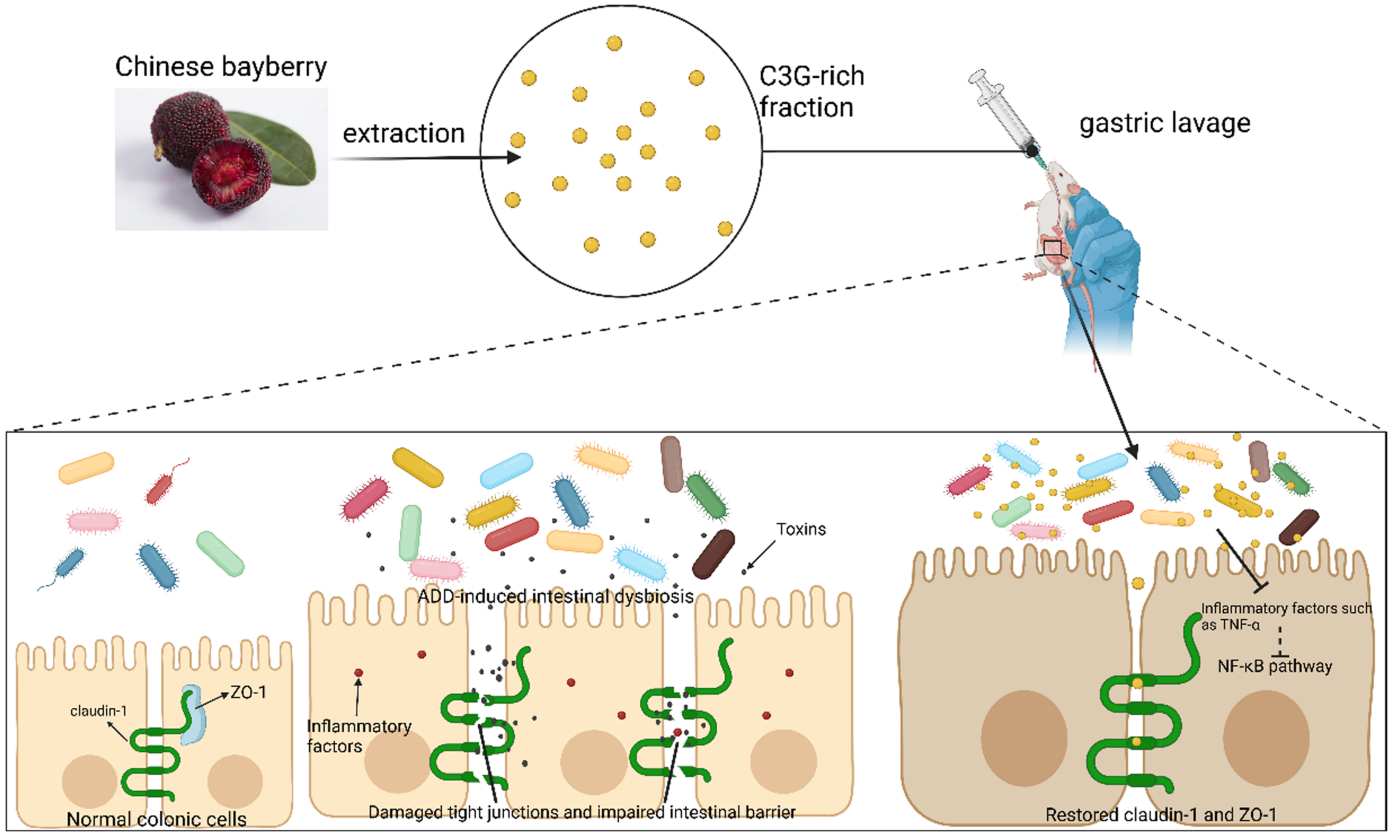
C3G reduces inflammation through gut flora. C3G alleviates inflammation by increasing the relative abundance of gut microbiota, which improves the intestinal environment. In conjunction with its metabolite protocatechuic acid, C3G enhances the expression of tight junction proteins in the intestinal epithelium, thereby reducing intestinal permeability and restoring the mucosal barrier, ultimately exerting therapeutic effects on the AAD mouse model.

In a mouse model of AAD established with lincomycin through gavage with C3G extracted from *Prunus Amygdalus*, C3G was found to treat diarrhea by positively regulating intestinal flora, decreasing inflammation, and recovering the intestinal mucosal barrier (Figure 4). High-throughput 16S sequencing of mouse intestinal contents revealed a significant increase in the percentage of *Anaplasmodial bacilli*, which can regulate the intestinal microenvironment in the intestines of mice treated with C3G; *Enterococcus faecalis*, which produces a wide range of toxicity factors contributing to the adherence, colonization, and infiltration of host tissues and modulates host immune, extracellular enzyme and toxin production; and *Enterococcus faecalis* (35), which can proliferate in intestinal inflammatory diseases (e.g., IBD and IBS). The relative abundance of *Clostridium difficile* was remarkably lower than that of the non-therapeutic group. This experiment also revealed that protocatechuic acid, a C3G metabolite, could reduce intestinal permeability and restore intestinal barrier functionality by adjusting the level of expression of ZO-1 fast-connecting proteins (Figure 4). Moreover, C3G maintains the intestinal mucosal boundary function by reducing the expression of inflammatory factors including TNF-*α* and enhancing the expressions of tight junction proteins regarding claudin-1 and ZO-1 (36).

#### 3.2.2 C3G causes inflammation by affecting adipose tissue

**Figure 5:**
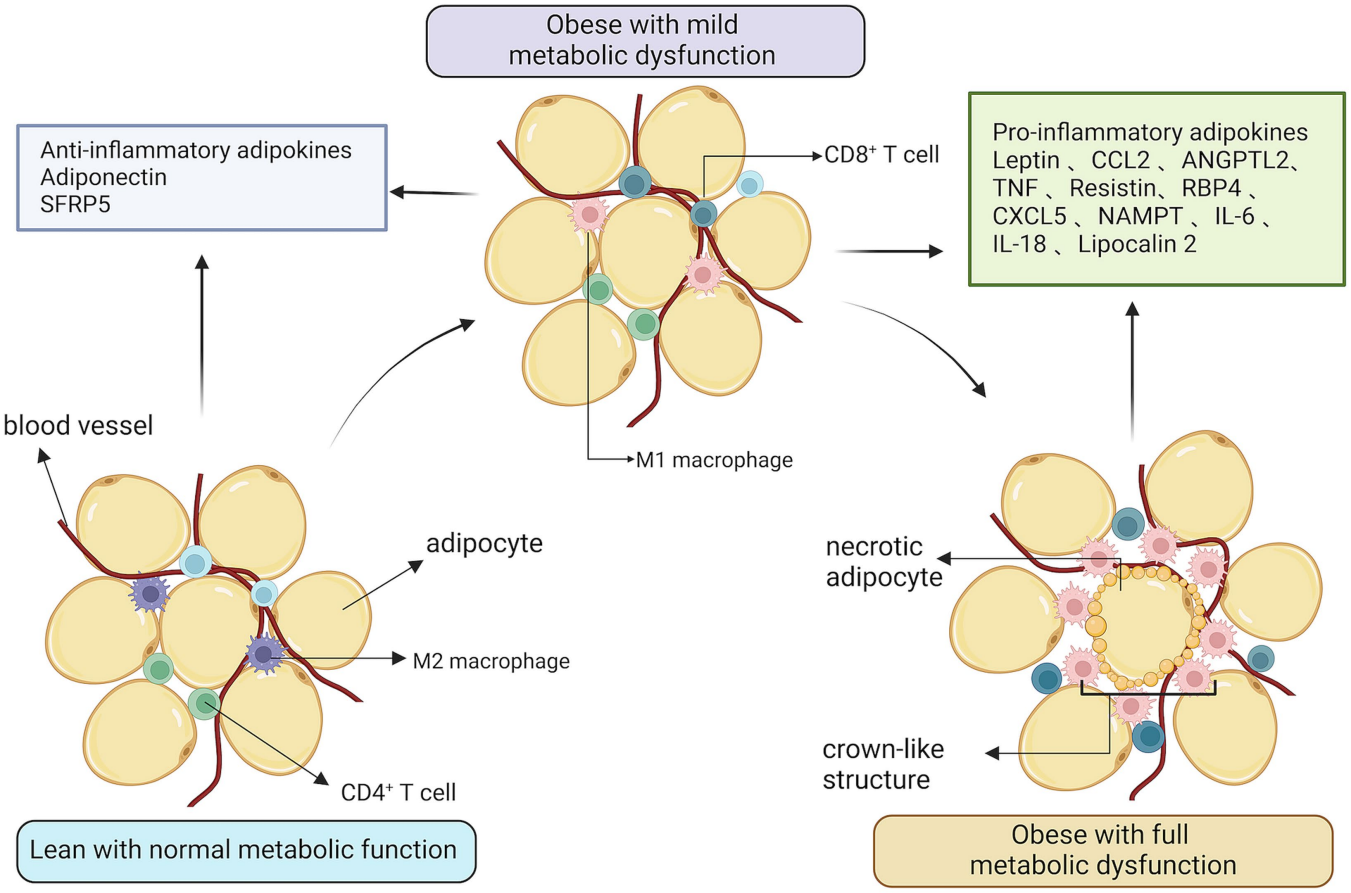
The relationship between obesity and inflammation. Expanded adipose tissue stroma results in the recruitment and accumulation of activated macrophages and T-cells. In an obese state, adipose tissue produces large amounts of pro-inflammatory factors and vascular function is reduced.

Immune cells, especially monocytes and macrophages, are aggressive participators in obesity-induced institutions and the complication of inflammation (37). The infiltration of adipose tissue by large numbers of macrophages adds to the number and activated state of the macrophages in adipose tissue and thus plays an important role in obesity-induced adipose tissue inflammation. With limited obesity, tissues may maintain a comparatively normometabolic function, with lower levels of immune cell activity and ample vascular functionality. Nevertheless, a mass change in enlarged adipose tissue could promote a shift toward a metabolic dysfunction phenomenon. While macrophages from lean adipose tissue express M2 or markers of selective activation of the state, obesity contributes to the collection and accumulation of M1 or classic active macrophages, along with T-cells, in adipose tissue. Inflammatory adipokines, which include lipocalin and secreted fold-associated protein 5, are produced with a preference for lean adipose tissue. In the obese state, adipose tissue produces a high number of pro-inflammatory agents, such as Il-6, MCP-1, iNOS, MMPs, and lipocalin (38) (Figure 5). Macrophage- associated pneumonia in white adipose tissue can therefore be attenuated by reducing the abundant abundance of pro-inflammatory gut microorganisms.

In addition, obesity increases the transcription of active, nuclear-localized NF-kB and as well as NF-kB target genes from the liver and skeletal muscle. NF-kB is a multiprotein transit factor with regulated targets that include secreted pro-inflammatory proteins such as TNF-a and MCP-1 (39). The targeted deficiency of I-kappa-B kinase, the kinase primarily responsible for NF-kB activity in the hepatocyte, decreases obedience-induced hepatic inflammation (e.g., like Il-6 and Il-1b) and circulation concentrations of the proinflammatory cytokines Il-1b and Il-6 (40). In a mouse model of obesity with the Ikbkb target mutation, mice with reduced hepatic and systemic inflammation, which would have also occurred, were found to have reduced insulin resistance (41). The pharmacologic inhibition of NF-kB was found to ameliorate insulin resistance using high doses of salicylic acid in an obese mouse model (42). Similar to the effect on NF-kB, being obese increased the activities of the JNK family of kinases from the liver, muscle, and adipose tissue, whereas pretreatment with C3G attenuated JNK phosphorylation (43). JNK kinases, which consist of three structure-related serine or threonine kinases, are part of the mitogen-activated protein kinase series, which are important proteins present in all cells that respond appropriately to stress (44). Therefore, obesity may be one of the causes of induced AS.

Excess circulation of foreign fatty acids has been shown to be a potential link to obesity (11). In this context, inhibition of lipolysis is a major goal to reduce foreign fatty acids and improve insulin susceptibility. Some studies have shown that C3G can inhibit the release of foreign fatty acids and glycerol in 3 T3-L1 adipocytes at high glucose. In 3 T3-L1 adipocytes, anthocyanins in colorful maize can block adipocyte polarization or lipid accumulations, and attenuate the PPAR-*γ* transcription (45). C3G has a regulatory effect on adipocyte differentiation. It promotes the programming of brown adipose tissue, reduces white adipose tissue, and decreases white fat weight, C3G-treated 3 T3-Ll cells differentiate to be smaller, insulin-insensitive adipocytes, and induces activation on the skeleton muscle metabolism. In addition, C3G up-regulated PPARγ in addition to C/EBP*α* gene expression (11). In addition, C3G up-regulates PPARγ and well as C/EBPα gene expression, resulting in an increase in lipocalin production, a decrease in TNF-α production, the activation of insulin signaling, and an increase in glucose ingestion (46).

### 3.3 Direct effects of C3G on inflammation

In Pratheeshkumar’s experiments, leukocyte infiltration, which is inflammation, was induced by irradiation with UVB. After the topical application of C3G, the dorsal skin of UVB-irradiated mice was sectioned from the UVB-irradiated skin to the UVB-unirradiated skin and then stained using hematoxylin eosin staining. Myeloperoxidase activity (MPO) served as a UVB-induced skin infiltration marker (6). The level of UVB-induced MPO was found to be higher in the skin of C3G-treated mice than that of non-C3G-treated mice. Thus, it can be concluded that C3G has an inhibitory effect on leukocyte infiltration and inflammation. However, in this experiment, how C3G could have an inhibitory effect on inflammation.

**Figure 6:**
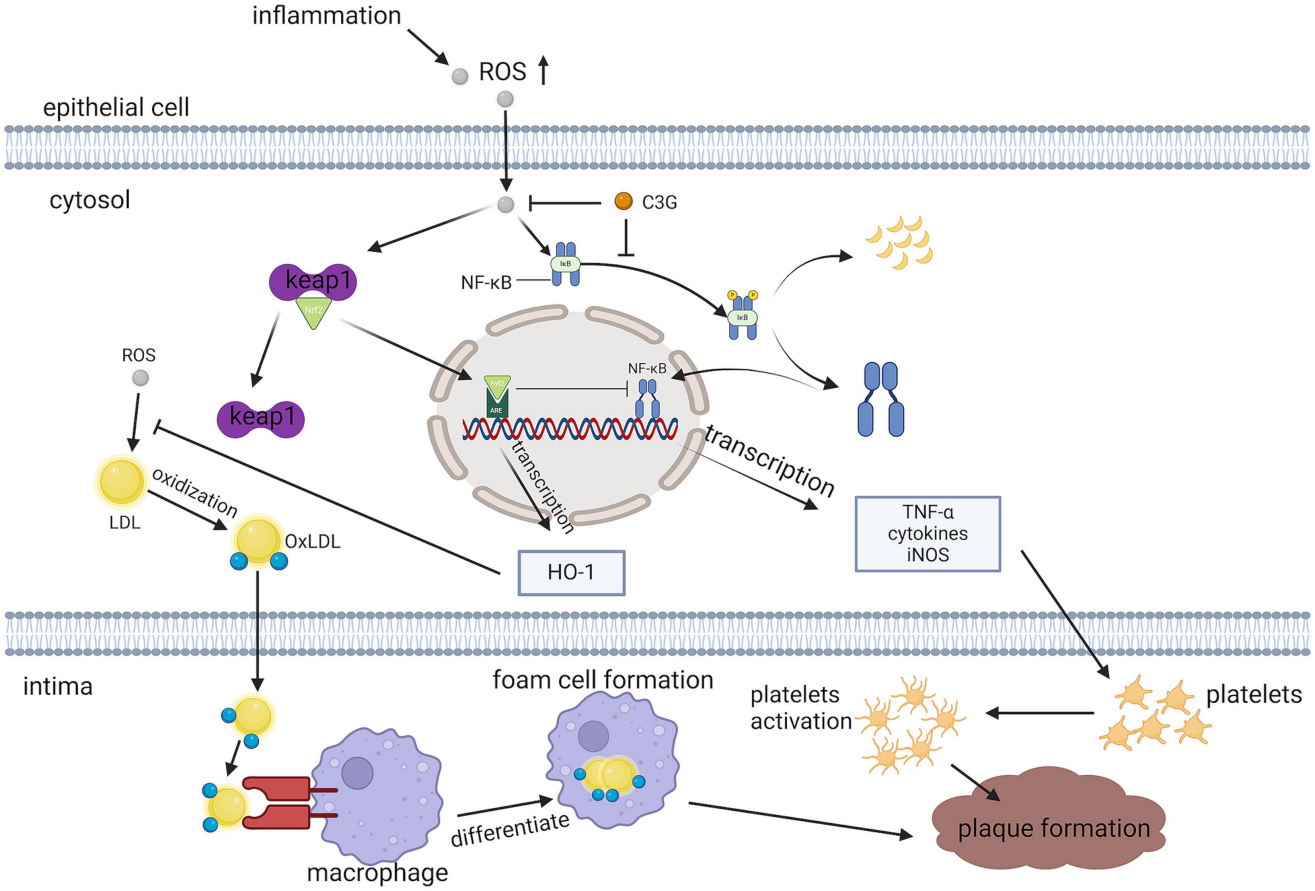
Pathways through which C3G reduces inflammation. C3G can attenuate LPS-induced pro-inflammatory cytokine production, and empty plaque formation, by inhibiting the activation of the NF-κB pathway and p65 translocation.

The THP-1 macrophage model is commonly used to study the anti-inflammatory actions of compounds. THP-1 cells were incubated with phorbol 12-myristate 13-acetate (40 ng/mL) for 48 h, to differentiate into macrophages (47). Lipopolysaccharide (LPS) induce the release of pro-inflammatory mediators from macrophages. They are endotoxins commonly present in the bacterial membranes of Gram-negative bacteria that induce multiple oxidative stresses, inflammatory markers, interleukin synthesis, and secretion (48). Under the stimulation of LPS, i-κB *α* is phosphorylated by its kinase IKK. This leads to its own ubiquitination and proteasomal degradation, which activate NF-κB and transports it into the nucleus, inducing the production of inflammation-associated genes (49). This can also reduce AS in apoE^−/−^ mice through ABCA1- and ABCG1-dependent cholesterol spillover in THP-1 macrophage-derived foam cells (50) (Figure 6).

Hao showed the mechanism of action of C3G by treating THP-1 macrophages with C3G and C3G liposomes and subjecting them to protein immunoblotting analysis (i.e., Western blot) to detect intracellular IκB-*α*, p-IκB-α, p65, and p-p65 content, and immunofluorescence to visualize the NF-κB p65 substrate The results of the nuclear translocations of NF-κB p65 subunits revealed that LPS significantly increased pro-inflammatory cytokine secretion, whereas cytokine secretion dose-dependently decreased under C3G and C3G liposome treatments. Cytokine secretion was better reduced by the C3G liposomes. In addition, the molecular mechanism of the anti-immune response of C3G or C3G liposomes was found to be related to the NF-*κ*B signaling channel, possibly by inhibiting phosphorylated proteins to limit the activity of the NF-κB channel and inhibiting the P65 translocation to limit the expressions toward the NF-κ b-related gene, IL-1β, IL-8, TNF-a, and IL-6 and achieve the purpose of alleviating inflammation (47).

## 4 C3G and vascular endothelium

The vascular endothelium is a specialized epithelial cell that exists between the vascular tissue and the plasma (13). Endothelial cells play an important role in vascular homeostasis by secreting many mediators such as nitric oxide (NO), endothelin (43), and prostacyclin (51), which modulate vascular tone, blood platelet activities, and clotting agents, and also affect vascular inflammation and cells migration. Clinicopathologic examination revealed significant endothelial cell structural and functional damage in patients with AS, leading to endothelial dysfunction. The possible reasons for endothelial dysfunction that contributes to AS, include raised and altered LDL; smoking-, hypertension-, and diabetes-induced free radicals; gene modifications; elevated plasma homocysteine plasma levels; contagious microorganisms; and combinations of these and other elements. Regardless of the reason for endothelial dysfunction, AS causes a heightened signature of reaction in specific arteries. Injury-induced endothelial malfunction resulting in a reparatory reaction can change the normal steady-state characteristics of the endothelium (52).

However, persistent inflammation increases the number of macrophages and lymphocytes (53), both of which migrate out of the circulation and multiply inside the location of the lesion. These cell activations result in the liberation of hydrolyzing enzymes, cytokines, chemokines, and growth elements, thus causing additional damage and, ultimately, focal necrosis. Consequently, a cycle of individual nucleated cell accumulation, smooth muscle cell migration and multiplication, and fibrous tissue formation leads to greater expansion and reorganization of the lesion. This leads to the lesion being covered by a fibrous cap that encloses the nucleus of the lipids and the necrotic tissue in what is known as a late-stage, complex lesion (54). To some extent, the arteries cannot compensate any longer by expanding, and the disease may invade the cavity and modify blood circulation.

### 4.1 Endothelial nitric oxide synthase (eNOS) activity and NO bioavailability

Endothelial dysfunction is characterized by reduced eNOS activity and NO bioavailability (55). NO from endothelial cells vasodilates blood vessels, inhibits platelet adhesion and aggregation, inhibits leukocyte-endothelial adhesion, and inhibits smooth muscle cell proliferation (56). Conversely, superoxide produced by oxidative stress rapidly inactivates NO and forms peroxynitrite. The formation of peroxynitrite may be another enhancement of signaling messengers to disrupt the bioavailability of NO (57). NOS catalogs the synthetic synthesis of NO from arginine, and it has three isoforms including eNOS (58).

C3G enhances endothelial cell migration and survival by increasing eNOS phosphorylation and maintaining the effectiveness of NO. In addition, C3G ameliorates the loss of endothelial progenitor cell function and endothelial repair, as endothelial cells derived from endothelial progenitor cells replace apoptotic endothelial cells (59), thereby slowing AS. C3G prevents and reverses the hypercholesterolemia-induced endothelial malfunction by suppressing the accumulation of cholesterol and the subsequent decrease in the production of hyperoxides in the aorta (1), thus maintaining eNOS activities and NO bio-availability.

C3G also exerts antioxidant effects through the up-regulation of Nrf2/ Ho-1, which was found to significantly increase the expression level of the transcription factor Nrf2 in the nucleus of the cells in an experiment pretreated with human anthocyanin drug serum to protect human umbilical vein endothelial cells (HUVECs) against TNF -*α*-induced damage improving antioxidant systems and activating the Nrf2/ARE pathway (43) (Figure 6).

In the context of AS, SIRT1 has been demonstrated to protect against DNA damage in human and rodent vascular cells (60). By contrast, decreased endothelial exposure to SIRT1 with aging may contribute to genetic group destabilization. Endothelial-specific SIRT1 overexpression leads to elevated eNOS expression (57). SIRT1 also enhances eNOS enzyme activity through denitrification. In endothelial cells, eNOS is specifically associated with SIRT1. The knockdown or inhibitory activities of SIRT1 promote the deacetylation of eNOS in the calmodulin- bound domains of lysine 496 and residue 506 (61). C3G, an atherogenic anthocyanin exhibiting antioxidant properties, has been demonstrated to increases the expressions of SIRT1 in a dose and a time dependent manner. Ota et al. reported that enhanced SIRT1 expression and physical coupling with eNOS ameliorated endothelial aging (62). Therefore, the SIRT1 -eNOS -NO axis is neuroprotective for endothelial malfunction and aging (57).

### 4.2 ABCG1 pathway

**Figure 7:**
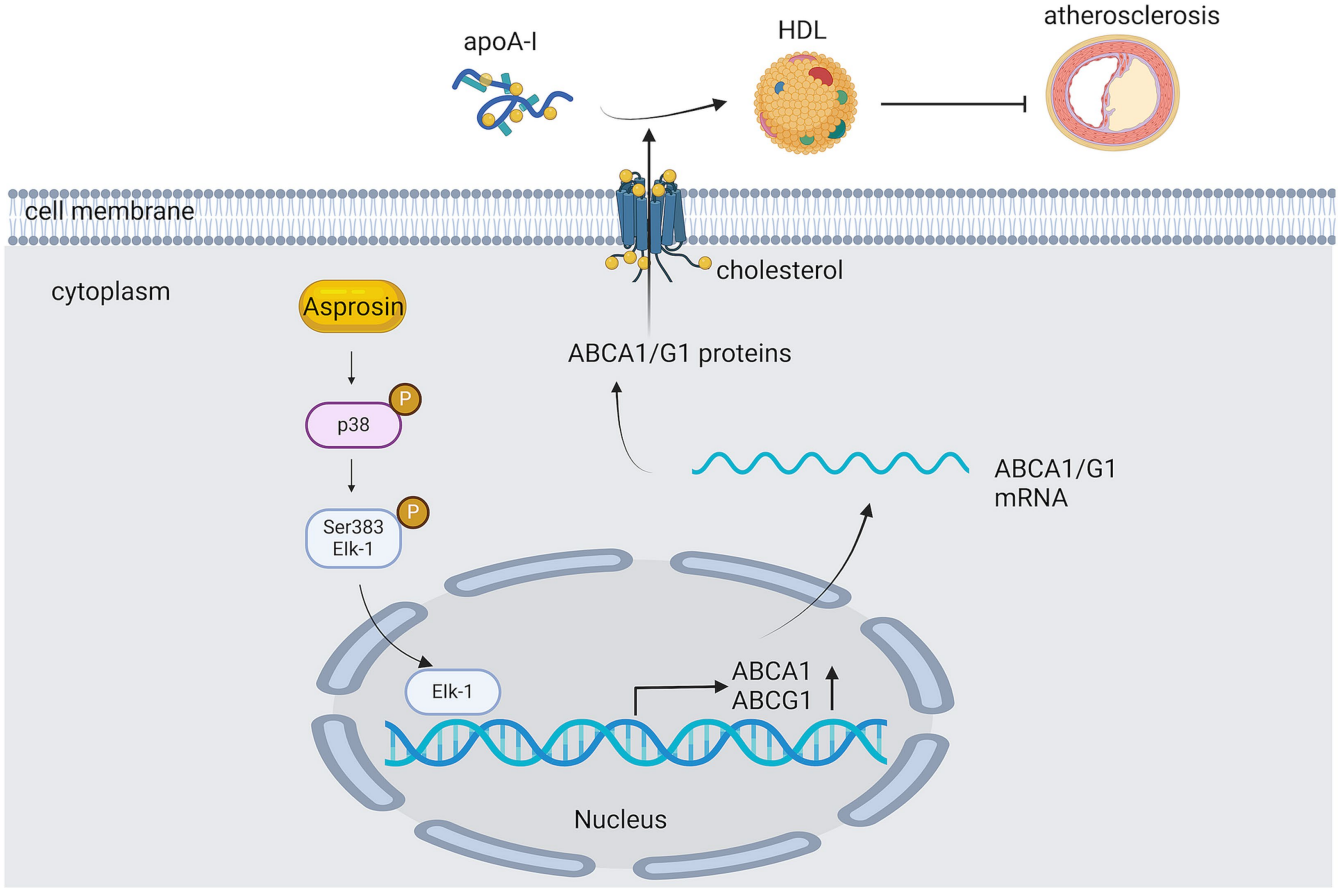
The role of ATP-binding cassette transporter protein G1 in AS. Asprosin inhibits macrophage lipid accumulation and reduces the atherosclerotic load by up-regulating ABCA1 and ABCG1 expression through the p38/Elk-1 pathway.

ABCG1 is part of the ABC caspase family of transporter protein and has been shown to modulate cellular fat turnover and promote cholesterol efflux in an autocrine (50) or para-secretory manner (63). ABCG1 is a member of the ATP-binding cassette transporter family and has been shown to regulate cellular cholesterol homeostasis by facilitating cholesterol efflux to HDL (50) (Figure 7). ABCG1 is also abundantly represented in endothelial cells and maintains endothelial functionality in mice on a high-cholesterol diet by facilitating the exocytosis of cholesterol and 7-ketocholesterol (55). In an experiment investigating the protection of C3G against hypercholesterolemia-induced endothelial dysfunction in apoE-deficient (apoE^2/2^) mice, C3G-fed mice were found to have higher expressions of ABCG1 and lower concentrations of cholesterol and 7-KC by comparing them with high-fat-cholesterol-rich diet mice (64); However, increased superoxide emission and generation over the course of hypercholesterolemia is a critical factor in the pathogenesis of endothelial function dysfunction and AS. This demonstrates that C3G can prevent or reverse hypercholesterolemia- induced endothelial dystrophy through the suppression of the accumulation of cholesterol and 7-oxosterol and the subsequent reduction of superoxide generation in the aorta, thus maintaining eNOS activities and the bioavailability of NO.

## 5 Conclusion

This review describes the effects on and the underlying mechanisms of C3G on AS by affecting intestinal microorganisms. C3G is a natural antioxidant and anti-inflammatory compound, and no reported side effects have been observed with anthocyanin drugs. It has been shown to lower blood pressure and blood glucose levels, improves lipid metabolism, decrease body mass, and mitigate multiple sclerosis; thus, it is highly utilized. Despite being one of the most abundant anthocyanins, C3G’s low bioavailability presents a challenge, as its concentration in the bloodstream remains minimal even after substantial intake. However, advancements such as nanoliposome encapsulation or acetylation have shown promise in significantly enhancing its bioavailability, laying a foundation for the therapeutic use of C3G in cardiovascular diseases, including AS, and intestinal dysbiosis.

When studying the mechanism of action of cornflower-3-glucoside, the relevant experimental data and mechanism of action were modeled on apolipoprotein E-deficient mice or rabbits as a model for AS. However, there are remarkable differences in the composition of the gut microbiome and the pathophysiology of cardiovascular diseases between humans and mice or rabbits, and there may be a difference between humans and mice in terms of the uptake and digestion, absorption of C3G. Therefore, whether the data collected in experimental animals can be directly generalized to humans must be established in the future. Despite these considerations, the therapeutic efficacy of C3G in AS is undisputed, offering a novel therapeutic avenue for the treatment of AS.

## Glossary

C3G: Cyanidin-3-glucoside
AS: Atherosclerosis
CVD: Cardiovascular Disease
IL: Interleukin
TNF-*α*: Tumor Necrosis Factor-alpha
eNOS: Endothelial Nitric Oxide Synthase
NO: Nitric Oxide
LDL: Low-Density Lipoprotein
HDL: High-Density Lipoprotein
ROS: Reactive Oxygen Species
SCFAs: Short-Chain Fatty Acids
NF-Κb: Nuclear Factor Kappa B
LPS: Lipopolysaccharide
PPAR-γ: Peroxisome Proliferator-Activated Receptor-gamma
ZO-1: Zonula Occludens-1
ABCG1: ATP-Binding Cassette Sub-Family G Member 1
TLRs: Toll-Like Receptors
SIRT: Sirtuin 1
MPO: Myeloperoxidase
JNK: c-Jun N-terminal Kinase
CRP: C-Reactive Protein
IKK: I-kappa-B kinase
IkB-α: Inhibitor of NF-κB alpha
ARE: Antioxidant Response Element
Nrf2: Nuclear Factor Erythroid 2-Related Factor 2
MAPK: Mitogen-Activated Protein Kinase
HO-1: Heme Oxygenase-1
AP-1: Activator Protein 1
TMAO: Trimethylamine N-oxide
IL-1β: Interleukin-1 beta
MCP-1: Monocyte Chemoattractant Protein 1
THP-1: Human Acute Monocytic Leukemia Cell Line
ABC: ATP-Binding Cassette
PCA: Protocatechuic Acid
FAs: Fatty Acids
AMPK: AMP-Activated Protein Kinase
HCD: High-Cholesterol Diet
MMPs: Matrix Metalloproteinases
ICAM-1: Intercellular Adhesion Molecule 1
VCAM-1: Vascular Cell Adhesion Molecule 1
TGF-β: Transforming Growth Factor Beta
EPC: Endothelial Progenitor Cell
SREBP-2: Sterol Regulatory Element-Binding Protein 2
HDAC: Histone Deacetylase
